# Magnetic resonance imaging features for differentiating breast papilloma with high-risk or malignant lesions from benign papilloma: a retrospective study on 158 patients

**DOI:** 10.1186/s12957-018-1537-9

**Published:** 2018-12-17

**Authors:** Li-Jun Wang, Ping Wu, Xiao-Xiao Li, Ran Luo, Deng-Bin Wang, Wen-Bin Guan

**Affiliations:** 1 Department of Radiology, Xinhua Hospital, Shanghai Jiao Tong University School of Medicine, No.1665, Kongjiang Road, Shanghai, 200092 China; 2 Department of Surgery, Xinhua Hospital, Shanghai Jiao Tong University School of Medicine, No.1665, Kongjiang Road, Shanghai, 200092 China; 3 Department of Pathology, Xinhua Hospital, Shanghai Jiao Tong University School of Medicine, No.1665, Kongjiang Road, Shanghai, 200092 China

**Keywords:** Breast, Papilloma, Magnetic resonance imaging, Differential diagnosis

## Abstract

**Background:**

Benign breast papilloma is currently managed with conservative management with close observation. In contrast, papilloma with high-risk or malignant lesions warrants surgical excision. The purpose of our study was to investigate magnetic resonance imaging (MRI) features of breast papilloma and to identify imaging diagnostic indicators for papilloma with high-risk or malignant lesions.

**Methods:**

MRI features of 175 surgically confirmed papillomas on 158 patients were retrospectively reviewed. The 175 cases included 132 cases of benign papilloma and 43 cases of papilloma with high-risk or malignant lesions. The MRI features of these lesions were classified into three types: mass, non-mass enhancement (NME), and occult lesion. The occult lesion was defined as the presence of only ductal dilation without any enhanced lesions on MRI. For a mass lesion, the mixed mass-NME lesion was considered if linear, segmental or regional enhanced lesion was found adjacent to the mass. Clinical and MRI features were compared by univariate and multivariate analysis between the benign papilloma and the papilloma with high-risk or malignant lesions.

**Results:**

Multivariate logistic regression analysis demonstrated that clinical characteristics including being or older than 50 years (odds ratio [OR] = 4.506), having bloody nipple discharge (OR = 4.499), and concurrent breast cancer (OR = 5.083) were significant indicators for papilloma with high-risk or malignant lesions. On MRI, most papillomas presented as mass (*n* = 135, 77.1%), and fewer as NME (*n* = 37, 21.1%) and occult lesion (*n* = 3, 1.7%). For the mass lesion, the logistic regression analysis demonstrated that a mass size exceeding 10 mm (OR = 2.956) and mixed mass-NME lesion (OR = 4.143) were independent risk indicators for a papilloma with high-risk or malignant lesions. For the NME lesion, the segmental or regional distribution was more commonly observed in the papilloma with high-risk or malignant lesions (61.5%) than the benign papilloma (12.5%) (*P* = 0.006). All the cases of occult lesions were benign papillomas.

**Conclusions:**

MRI features including a mass size exceeding 10 mm, mixed mass-NME lesion, and NMEs with segmental or regional distribution indicate a papilloma with high-risk or malignant lesions.

## Introduction

Breast intraductal papilloma is characterized by a finger-like fibrovascular core lined by epithelial and myoepithelial cells [1]. It could coexist with a broad spectrum of benign, high-risk, and malignant lesions including benign proliferative lesions, atypical hyperplasia, lobular neoplasia, ductal carcinoma in situ (DCIS), and even invasive carcinoma [1–3]. The clinical management and outcome of intraductal papillomas with different kinds of lesions differ. Benign papilloma with or without benign proliferative lesions is managed conservatively with close observation [4]. In contrast, papilloma with malignant lesions warrants complete surgical excision [5–7]. Papilloma with high-risk lesions, which is associated with a significant risk of breast cancer, is also recommended to complete surgical excision [5, 7, 8]. Therefore, it is essential to differentiate between benign papilloma and papilloma with high-risk or malignant lesions.

It is a challenge for a pathologist to differentiate between benign papilloma and papilloma with high-risk or malignant lesions with the use of limited tissues obtained from core needle biopsy (CNB) [6, 7]. CNB was reported to have an underestimation rate of 0% to 33% in diagnosing papillary breast lesions [6, 7]. Currently, there is a consensus that papilloma with high-risk or malignant lesions diagnosed on CNB requires subsequent surgical excision [6]. However, the management of benign papilloma diagnosed on CNB remains controversial. Some investigators recommended imaging follow-up [9–11]; in contrast, some authors suggested surgical excision [7, 12, 13].

Studies have demonstrated that close imaging follow-up or surgical excision should be recommended for the benign papillomas diagnosed on CNB when there is a clinical or radiologic suspicious lesion [9, 14, 15]. Han et al. [14] enrolled 383 cases of benign papillomas diagnosed on CNB and evaluated the upgrading rate after excision. They found that no case was underestimated when the cases with suspicious clinical symptoms were excluded. Wen [9] suggest 3 to 6 months follow-up for patients with benign papillomas diagnosed on CNB if there were suspicious radiologic findings. Moritani et al. [15] strongly recommended surgical excision for papilloma in CNB when there was a radiologic segmental abnormality because this might indicate a coexisting DCIS. Therefore, evaluating the clinical and imaging risk factors which indicate papillomas with high-risk or malignant lesions helps to guide the clinical management of breast papillomas.

Mammography and ultrasound were reported not to be able to differentiate benign from malignant papillary lesions [12]. Breast magnetic resonance imaging (MRI) is currently regarded as the most sensitive modality in detecting breast lesions [16]. MRI was reported to have a higher sensitivity in defining the number and the extent of the papillary lesions than mammography and ultrasound [17–19]. Several studies described the MRI features of papilloma with a sample size ranging from 18 to 83 [18, 20–26]. However, most of these studies focused on the MRI features of benign papillomas, and only one study involved the differential diagnosis between benign and papilloma with malignant lesions [20]. The MRI features of papillomas with high-risk or malignant lesions and the differential diagnosis between benign papillomas and papillomas with high-risk or malignant lesions remain unclear. Therefore, we designed the present study to investigate MRI features of breast papillomas and to identify clinical and MR imaging risk indicators for papillomas with high-risk or malignant lesions.

## Methods

### Patients

This retrospective study was approved by the Institutional Review Board of our Hospital, and the informed consent requirement was waived (approval no. XHEC-D-2015-153). Between September 2012 and August 2015, 238 patients who had intraductal papillomas confirmed by surgically excisional biopsy and preoperative MRI assessment in our hospital were retrieved from our database. The microscopic papillomas which were excised because of other breast lesions outside the papilloma and incidentally discovered by the pathologists were excluded. Among the 238 patients, the 81 patients with 87 microscopic papillomas which presented no abnormal findings on breast MRI or hardly be recognized from the background parenchymal enhancement were excluded. There was one patient who had a microscopic papilloma in one side being excluded and a papilloma in another side being enrolled. Finally, 158 patients with 175 papillomas (17 patients have bilateral lesions) were enrolled.

The indications of the 175 cases for MRI were pathological nipple discharge with or without palpable mass (*n* = 56), only palpable mass (*n* = 54), suspicious lesions on screening ultrasound (*n* = 31), and other palpable mass outside the papilloma (*n* = 34). Preoperative Breast Imaging Reporting and Data System (BI-RADS) categories of these lesions were reviewed and the highest BI-RADS category of mammography, ultrasound, and MRI was recorded. Thirty-four of 175 lesions had a BI-RADS 3 category, and 141 lesions had a BI-RADS 4 or 5 category. Of the 34 cases who had a BI-RADS 3 category, 15 cases were diagnosed as papillomas or intraductal lesions and then biopsied, and the other 19 cases which were diagnosed as other kinds of benign lesions were also treated with biopsy instead of follow-up. All the 141 suspicious lesions with a BI-RADS 4 or 5 category were biopsied. Among the 175 lesions, 114 underwent lumpectomy, 56 underwent segmentectomy of the dilated duct, and 5 underwent quadrantectomy. Mastectomy or nipple sparing mastectomy was performed in eight patients with DCIS component after the excisional biopsy. The clinical demographics of the enrolled cases including the patients’ age, menopause status, bloody nipple discharge, personal history of papillary breast lesion and breast cancer, concurrent breast cancer (contralateral or ipsilateral), and family history of breast cancer were systematically retrieved.

### Imaging techniques

Imaging was performed on a 3.0-T whole-body MRI scanner (Signa HDxt; GE Healthcare). The patients were positioned in the prone position with both breasts placed in an eight-channel phase-array breast coil. Firstly, axial short tau inversion recovery (STIR) sequence was obtained (TR/TE/TI, 7060/35.2/170 ms; slice thickness, 4 mm; gap, 1 mm; matrix, 320 × 192, FOV, 32 cm). Next, axial diffusion-weighted imaging (DWI) (*b* = 0, 800 s/mm^2^; TR/TE, 5125/66.4 ms; slice thickness, 4 mm; gap, 1 mm; matrix, 128 × 128, FOV, 32 cm) was performed in 165 patients. Volume Image Breast Assessment (VIBRANT) sequence (TR/TE/TI, 4.3/2.1/14 ms; slice thickness, 1.2 mm; gap, 0 mm; matrix, 416 × 320, FOV, 42 cm) were obtained before and at 54 s, 108 s, 162 s, 216 s, and 270 s after the intravenously injection of Gadopentetate Dimeglumine (Gd-DTPA; Beilu, Beijing, China) with 0.1 mmol/kg at a flow rate of 2 mL/s and 20-mL normal saline flush.

### Image analysis

One radiologist with 7-year experience in breast MRI confirmed the location of the papillary breast lesions on MRI according to preoperative ultrasonography localization reports, MRI reports, surgical records, and the pathology results of the patients from the medical records system. Then, MRI features of all the lesions were analyzed by two trained breast radiologists with more than 4 years of experience in consensus. The disputes were resolved via consultation with a third experienced breast radiologist with over 20 years of experience. The radiologists were blinded to the clinical symptoms and histopathology.

The MRI features of papillomas were evaluated according to the fifth BI-RADS atlas [27]. The lesions were classified into three types as follows: mass, non-mass enhancement (NME), and occult lesions. Analysis of the breast with multiple masses was conducted using the imaging features of the largest mass on MRI. The shape of the mass was classified as oval, round, or irregular [27]. The margin of the mass was classified as circumscribed or non-circumscribed [27]. The masses were grouped into three as solid, complex cystic, or cystic masses. The presence of accompanying NME to mass lesions was also evaluated. The mixed mass-NME lesions were recorded if linear, segmental, or regional enhancement was found adjacent to the mass. The occult lesion was defined as the presence of only ductal dilation without any enhanced lesions on MRI.

The size of the lesion was measured on subtracted axial images of the second series as maximum tumor diameter. The time-signal intensity curve (TIC), apparent diffusion coefficient (ADC) value, and maximum intensity projection (MIP) were obtained on a workstation (GE Healthcare). The TIC was obtained from the dynamic sequence. Oval or round regions of interest (ROIs) for obtaining TIC were set on the lesion and avoided cystic regions and duct dilation areas. According to the BI-RADS atlas [27], the TIC patterns were classified as persistent, plateau, and washout. TIC patterns were available for 172 non-occult lesions. The ADC values were measured on ADC maps. Three 11–70 mm^2^ ROIs were set on the lesion without including cystic regions and duct dilation areas. The average value of three measurements was used as the ADC value. ADC values were available for 78 lesions. The reasons for missing ADC values in 97 cases were (1) the size of the lesion was too small to identify on DWI (*n* = 48), or (2) DWI failure (*n* = 42), or (3) a cystic mass with a very thin wall to place an ROI (*n* = 4), or (4) occult lesions (*n* = 3).

### Statistical analysis

Analyses were performed by using the SPSS 23.0 (IBM Corp.) analysis software package, and a *P* value less than 0.05 was regarded as statistically significant. According to the pathology results, the cases were classified into the group A (papilloma with or without benign proliferative lesions) and group B (papilloma with high-risk or malignant lesions). Student’s *t* test or Mann-Whitney *U* test was used to compare the quantitative variables between the two groups. Chi-squared test or Fisher’s exact test was employed for the comparison of the categorical variables between the two groups. Multivariate logistic regression analysis was employed to identify clinical or MRI diagnostic risk indicators for papilloma with high-risk or malignant lesions. The variables which showed *P* values less than 0.1 in the univariate analysis were selected for the multivariate analysis. The “Forward: likelihood ratio” mode was adopted. The probability threshold of entry and removal for stepwise is 0.05 and 0.1, respectively.

## Results

### Pathological and clinical characteristics results

The 175 cases of papillomas included 132 cases of intraductal papilloma, 22 cases of papilloma with atypical hyperplasia, and 21 cases of papilloma with DCIS. The clinical features of papillomas were summarized in Table 1. The mean age of patients in group B was 56.4 ± 11.6 years, and for those in group A, 47.4 ± 12.3 years (*P* <  0.001). Postmenopause and bloody nipple discharge were more commonly observed in group B than group A (*P* = 0.006 and *P* = 0.009, respectively). In two patients, synchronous breast cancer was found in the same breast outside the papilloma. In six patients, breast cancer was found in the contralateral breast. In one patient, bilateral breast cancers were confirmed outside the papilloma. Concurrent breast cancer was detected more often in group B (11.6%) than group A (3.0%) (*P* = 0.069). No significant difference was observed in the personal histories in these two groups. Multivariate logistic regression analysis demonstrated that being or older than 50 years (odds ratio [OR] = 4.506), having bloody nipple discharge (OR = 4.499), and concurrent breast cancer (OR = 5.083) were significant indicators for papilloma with high-risk or malignant lesions (Table 2).

**Table 1:** Clinical features of breast papillomas

Variables	Group A (*n* = 132)	Group B (*n* = 43)	*P* value
Age (years)	47.4 ± 12.3	56.4 ± 11.6	< 0.001
Age (years)			< 0.001
< 50	79 (59.8%)	12 (27.9%)	
≥ 50	53 (40.2%)	31 (72.1%)	
Postmenopause	51 (38.6%)	27 (62.8%)	0.006
Bloody nipple discharge	17 (12.9%)	13 (30.2%)	0.009
Personal history of a papillary lesion	3 (2.3%)	0 (0%)	1.000
Personal history of breast cancer	3 (2.3%)	1 (2.3%)	1.000
Concurrent breast cancer	4 (3.0%)	5 (11.6%)	0.069
Family history of breast cancer	2 (1.5%)	3 (7.0%)	0.180

**Table 2:** Multivariate analysis of clinical features between group A and B

Variables	OR	95% CI	*P* value
Age (years)			< 0.001
< 50	1		
≥ 50	4.506	2.003–10.136	
Bloody nipple discharge	4.499	1.784–11.345	0.001
Concurrent breast cancer	5.083	1.169–22.102	0.030

*OR* = odds ratio; *CI* = confidence interval

### MRI features of the lesions

On MRI, most papillomas presented as mass (*n* = 135, 77.1%), and fewer as NME (*n* = 37, 21.1%) and occult lesion (*n* = 3, 1.7%). Thirty cases (22.2%) of the mass lesions were papillomas with high-risk or malignant lesions. Thirteen cases (35.1%) of NME lesions were papillomas with high-risk or malignant lesions. All the three cases of occult lesions were benign papillomas, and the pathological findings revealed that the diameter of papillomas was 1 to 2 mm.

**Fig. 1:**
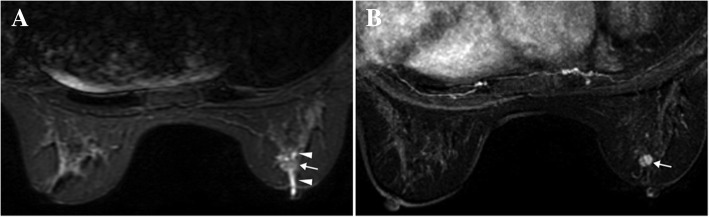
A case of papilloma manifesting as a solid mass (in 64-year-old woman). **a** STIR sequence reveals duct dilatation (arrowheads) and intraluminal filling defects (arrow) in the right breast. **b** The MIP of the subtracted image shows an enhanced solid mass with a smooth margin (arrow) in the area corresponding to the intraluminal filling defects area on STIR sequence. The pathology is an intraductal papilloma

**Fig. 2:**
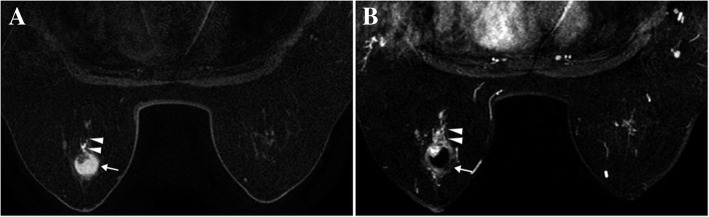
A case of papilloma with DCIS manifesting as a complex cystic mass (in 59-year-old woman). **a** The pre-contrast T1-weighted image reveals a mass (arrow) with nodular protrusion inside and duct dilation (arrowheads) in the left breast. **b** The MIP image of the subtracted images shows an enhanced complex cystic mass (arrow) and mixed mass-NME lesion (arrowheads). The pathology is intraductal papilloma with DCIS

The MRI features of mass lesions were summarized in Table 3. The median size of group B was 9.3 (7.6, 14.4) mm, which was larger than that of group A, 8.2 (6.2, 9.8)mm (*P* = 0.020). Papillomas more frequently presented as a solid mass (*n* = 121) (Fig. 1), and less frequently as complex cystic mass (*n* = 10) (Fig. 2) and cystic mass (*n* = 4) on MRI. Pathological findings revealed that all the cystic masses on MRI showed infarction of the papillary fronds. The presence of duct dilatation was more commonly seen in group B than group A (66.7% vs. 42.9%, *P* = 0.021). The mixed mass-NME lesion was also more frequently detected in group B than in group A (50.0% vs.19.0%, *P* = 0.001). No significant differences were found for the location, number of masses (being solitary or multiple), shape, margin, TIC pattern, and ADC value between the two groups. Multivariate logistic regression analysis demonstrated that a mass size exceeding 10 mm (OR = 2.956) and mixed mass-NME lesion (OR = 4.143) were significant indicators for papilloma with high-risk or malignant lesions (Table 4).

**Table 3:** MRI features of papillomas manifesting as mass lesions

Variables	Group A (*n* = 105)	Group B (*n* = 30)	*P* value
Size (mm)	8.2 (6.2–9.8)	9.3 (7.6–14.4)	0.020
Size (mm)			0.009
≤ 10 mm	84 (80.0%)	17 (56.7%)	
> 10 mm	21 (20.0%)	13 (43.3%)	
Location			0.782
Anterior third of the breast	52 (49.5%)	14 (46.7%)	
Middle or posterior third of the breast	53 (50.5%)	16 (53.3%)	
Number of mass			0.604
Solitary	65 (61.9%)	17 (56.7%)	
Multiple	40 (38.1%)	13 (43.3%)	
Shape			0.299
Oval or round	83 (79.0%)	21 (70.0%)	
Irregular	22 (21.0%)	9 (30.0%)	
Margin			0.267
Circumscribed	71 (67.6%)	17 (56.7%)	
Non-circumscribed	34 (32.4%)	13 (43.3%)	
Mass type			0.340
Solid mass	96 (91.4%)	25 (83.3%)	
Complex cystic mass	6 (5.7%)	4 (13.3%)	
Cystic mass	3 (2.9%)	1 (3.3%)	
Duct dilation	45 (42.9%)	20 (66.7%)	0.021
Mixed mass-NME lesion	20 (19.0%)	15 (50.0%)	0.001
TIC			0.623
Persistent	38 (36.2%)	8 (26.7%)	
Plateau	31 (29.5%)	10 (33.3%)	
Washout	36 (34.3%)	12 (40.0%)	
ADC value (× 10^−3^ mm/s^2^)*	1.33 ± 0.22	1.29 ± 0.13	0.565

*TIC* = time-signal intensity curve; *ADC* = apparent diffusion coefficient

*ADC values of 51 cases of benign papillomas and 12 cases of the non-benign papillomas were available

**Table 4:** Multivariate analysis of MRI features between group A and B manifesting as mass lesions

Variables	OR	95% CI	*P* value
Size			0.020
≤ 10 mm	1		
> 10 mm	2.956	1.190–7.345	
Mixed mass-NME lesion	4.143	1.702–10.088	0.002

*OR* = odds ratio; *CI* = confidence interval

**Fig. 3:**
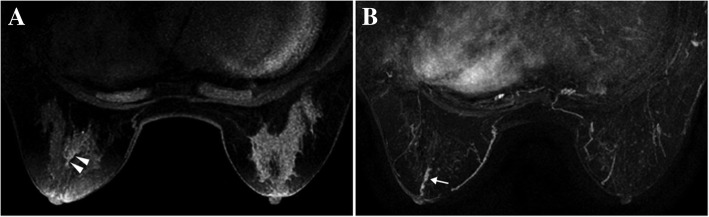
A case of papilloma manifesting as linear enhancement (in 52-year-old woman). **a** The MIP of the pre-contrast T1-weighted image reveals duct dilation (arrowheads) in the left breast. **b** The MIP of the subtracted image shows abnormal enhanced lesion with linear distribution (arrow). The pathology is intraductal papilloma with adenosis

**Fig. 4:**
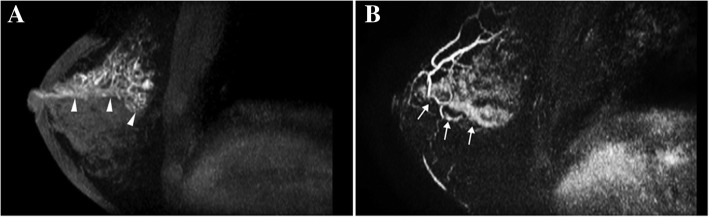
A case of papilloma with DCIS manifesting as segmental enhancement (in 53-year-old woman). **a** The MIP of the pre-contrast T1-weighted image reveals duct dilation (arrowheads) in the right breast. **b** The MIP of the subtracted image shows abnormal enhanced lesion with segmental distribution (arrows). The pathology is intraductal papilloma with DCIS

The MRI features of NME lesions were summarized in Table 5. For NME lesions, focal or linear distribution (Fig. 3) was observed more commonly in group A (87.5% vs. 38.5%); however, segmental or regional distribution (Fig. 4) was detected more often in group B (61.5% vs. 12.5%) (*P* = 0.006). No significant difference was found in size, duct dilation, TIC pattern, and ADC value between the two groups.

**Table 5:** MRI features of papillomas manifesting as NME lesions

Variables	Group A (*n* = 24)	Group B (*n* = 13)	*P* value
Size (mm)	20.9 (11.8–29.0)	27.3 (14.0–45.8)	0.294
Distribution			0.006
Focal or linear	21 (87.5%)	5 (38.5%)	
Segmental or regional	3 (12.5%)	8 (61.5%)	
Duct dilation	14 (58.3%)	11 (84.6%)	0.149
TIC			0.270
Persistent	16 (66.7%)	6 (46.2%)	
Plateau	6 (25.0%)	3 (23.1%)	
Washout	2 (8.3%)	4 (30.8%)	
ADC value (×10^−3^ mm/s^2^)*	1.32 ± 0.29	1.32 ± 0.23	1.000

*TIC* = time-signal intensity curve; *ADC* = apparent diffusion coefficient

*ADC values of eight cases of benign papillomas and seven cases of the non-benign papillomas were available

## Discussion

In this study, we found patients with papillomas with high-risk or malignant lesions were older than those with benign papillomas, which was consistent with previous reports [28, 29]. Our study indicated that the risk of a papilloma with high-risk or malignant lesions in a patient being or older than 50 years was 4.506 times that equal to a patient younger than 50 years. The bloody nipple discharge was more commonly observed in patients with papilloma with high-risk or malignant lesions (30.2%) than those with the benign papilloma (12.9%). The bloody nipple discharge occurred in the patient with benign papilloma may be caused by the hemorrhage of the tumor or duct ectasia [30]. In our study, bloody nipple discharge (OR = 4.499) was also proved to be a risk predictor indicating a papilloma with high-risk or malignant lesions. Han et al. [14] firstly reported that the presence of concurrent contralateral breast cancer was a predictive factor for papillomas diagnosed on CNB upgrading to malignancy. Our study supports this finding. Our results demonstrated that the concurrent contralateral or ipsilateral breast cancer outside the papilloma was an independent risk indicator for a papilloma with high-risk or malignant lesions (OR = 5.083).

On MRI, 77.1% of papillomas presented as the mass in our study. We found that a mass size exceeding 10 mm was a risk indicator for a papilloma with high-risk or malignant lesions, consistent with previous studies [28, 29]. Tominaga et al. [26] described four MRI types of benign papilloma as follows: oval nodule, irregular nodule, solid and cystic mass, and occult lesion. The irregular margin of benign papilloma correlated with the surrounding fibrosis or collagenization of the stroma of the lesion [26]. Our study revealed that most of the papillomas have round or oval shape and more than half of the non-benign papillomas have circumscribed margins. In this study, shape and margin were found as non-distinctive morphologic features between benign papilloma and a papilloma with high-risk or malignant lesions. The complex cystic pattern was slightly more commonly observed in a papilloma with high-risk or malignant lesions. In the present study, we found a small portion of papillomas manifested as a cystic mass on MRI. Pathological findings revealed the infarction of the papillary fronds in all these cases. To the best of our knowledge, this feature has not been reported in previous literature.

Studies have demonstrated that papillomas are usually mixed with benign proliferative lesions and less commonly with atypical and malignant lesions [3–9]. Lewis et al. [3] reported that 64% of single papilloma and 93% of multiple papillomas had concomitant ductal hyperplasia. Choi et al. [29] reported 65 in 182 (35.7%) cases of papilloma coexisted with atypical or malignant lesions. In our cases, 43 in 175 (24.6%) cases of papilloma had concomitant atypical or malignant lesions. We also found that mixed mass-NME lesion was more commonly observed in papilloma with high-risk or malignant lesions (50.0%) than the benign papillomas (19.0%). We speculated that the accompanying NME lesions on MRI may be attributed to the concomitant lesions, and the atypical hyperplasia or DCIS adjacent to the papilloma might more likely show enhancement than the benign hyperplasia lesions. The mixed mass-NME lesion (OR = 4.143) was proved to be an independent predictor for papilloma with high-risk or malignant lesions in this study.

On MRI, 21.1% cases of papilloma presented as pure NME in our study. Papilloma manifesting as NME could be due to the concomitant benign, atypical, and malignant proliferative lesions. Moritani et al. [15] considered that a lesion with a segmental abnormality in at least one breast imaging modalities indicated malignancy and should be surgically resected. Sarica et al. [20] found that the segmental enhancement was more frequently seen in papillomatosis and malignant papillary lesions than benign papillomas. Our study also demonstrated that the segmental or regional distribution indicated a non-benign papilloma.

Studies [23, 25] reported that 55.0–72.7% cases of benign papillomas showed washout curves. However, in our cases, 34.3% cases of the mass lesions and 8.3% cases of NME lesions had washout curves. The larger sample size may be one reason for the difference in TIC patterns. The concomitant proliferative lesions could also have an influence on the TIC patterns of papillomas. Our study showed that TIC patterns could not add any value for differential diagnosis. The ADC value of papilloma was reported as 1.17 ± 0.24 mm^2^/s at *b* value of 1000 s/mm^2^, similar to that of small invasive carcinoma (1.13 ± 0.18 mm^2^/s) [25]. In our cases, at *b* value of 800 s/mm^2^, the mean ADC value of papilloma was 1.33 mm^2^/s for the mass lesion. A relatively lower *b* value may be responsible for the slight higher ADC values of papilloma in our study. We also found that the papilloma with high-risk or malignant lesions showed similar ADC values with the benign papillomas without significant difference.

Tominaga et al. [26] described that the papillomas which manifested as only ductal dilation without detectable masses on MRI as occult lesions. This kind of papilloma was too small to be detected on enhanced MRI. It usually presents no abnormal findings on breast MRI or hardly be recognized from the background parenchymal enhancement. For patients with MRI-occult lesions, fiberoductoscopy could be a new problem-solving tool. Fiberoductoscopy enables to direct visualization of small lesions and performs minimally invasive procedures [30]. Therefore, it may help to reduce unnecessary surgical excision in patients with benign intraductal lesions [30]. More studies are needed to evaluate the subsequent management for fiberoductoscopy diagnosed benign papillomas in patients with pathological nipple discharge. In our study, all the three cases of occult lesions were benign. Moreover, the microscopic papillomas excluded in our study were also occult on breast MRI. Jaffer et al. [31] have demonstrated that the microscopic papilloma, which may not be clinically or radiologically evident, is very unlikely to be associated with malignancy and do not need to be excised.

The main limitation of this study is that the patients in this cohort underwent excisional biopsy instead of CNB. These patients usually had suspicious breast lesions diagnosed on mammography or ultrasound and then underwent preoperatively MRI assessment, ultrasound localization, and subsequently surgically excisional biopsy. The injection of methylene blue into the dilated ducts was performed for patients with nipple discharge before the surgical excision. Therefore, we could not assess the influence of MRI on the management of CNB diagnosed benign papilloma. Secondly, we did not compare the value of MRI with ultrasound in evaluating the extent of papillomas with pathologic correlation. Though there were limitations, we still could find some clinical and MR imaging diagnostic indicators for papillomas with high-risk or malignant lesions from this series of surgically confirmed papillomas. Our findings are applicable for patients who already had an MRI examination for a different reason, for example, screening high-risk women, or the evaluation of a suspected or a newly diagnosed breast cancer, and so on. If the patient was diagnosed as papilloma by CNB, the MRI diagnostic indicators reported in this study help to assist the surgeon for the subsequent management, surgical excision, or follow-up. A prospective study is warranted to assess the value of MRI in the management a patient with CNB diagnosed benign papilloma.

## Conclusions

In conclusion, for a patient with a papilloma, clinical features including being or older than 50 years, having bloody nipple discharge, and concurrent breast cancer were significant indicators for papillomas with high-risk or malignant lesions. On MRI, most papillomas presented as mass, and fewer as NME and occult lesion. For a papilloma, a mass size exceeding 10 mm with a mixed mass-NME pattern or NMEs with segmental or regional distribution on MRI were main indicators for a papilloma with high-risk or malignant lesions.

## Abbreviations

ADC: Apparent diffusion coefficient value
BI-RADS: Breast imaging reporting and data system
CI: Confidence interval
CNB: Core needle biopsy
DCIS: Ductal carcinoma in situ
DWI: Diffusion-weighted imaging
FOV: Field of view
MIP: Maximum intensity projection
MRI: Magnetic resonance imaging
NME: Non-mass enhancement
OR: Odds ratio
ROI: Region of interest
STIR: Short tau inversion recovery
TE: Echo time
TI: Inversion time
TIC: Time-signal intensity curve
TR: Repetition time
VIBRANT: Volume image breast assessment
